# The Use of Filled Pauses Across Multiple Discourse Contexts in Children Who Are Hard of Hearing and Children with Typical Hearing

**DOI:** 10.3390/bs15081053

**Published:** 2025-08-04

**Authors:** Charlotte Hilker, Jacob J. Oleson, Mariia Tertyshnaia, Ryan W. McCreery, Elizabeth A. Walker

**Affiliations:** 1 Department of Communication Sciences and Disorders, University of Iowa, Iowa City, IA 52242, USA; charlotte-hilker@uiowa.edu; 2 Department of Biostatistics, University of Iowa, Iowa City, IA 52242, USA; jacob-oleson@uiowa.edu (J.J.O.); mariia-tertyshnaia@uiowa.edu (M.T.); 3 Boys Town National Research Hospital, Omaha, NE 68131, USA; ryan.mccreery@boystown.org

**Keywords:** children, hearing loss, language samples, filled pauses

## Abstract

Filled pauses are thought to be reflections of linguistic processes (e.g., lexical retrieval, speech planning and execution). *Uh* may be a self-directed cue for when a speaker needs more time to retrieve lexical–semantic representations, whereas *um* serves as a listener-directed, pragmatic cue. The use of filled pauses has not been examined in children who are hard of hearing (CHH). Participants included 68 CHH and 33 children with typical hearing (CTH). Participants engaged in conversations, expository discourse, and fable retells. We analyzed filled pauses as a function of hearing status and discourse contexts and evaluated the relationship between filled pauses and language ability. CHH produced *uh* across discourse contexts more often than their hearing peers. CHH did not differ in their use of *um* relative to CTH. Both *um* and *uh* were used more often in conversational samples compared to other types of discourse. Spearman’s correlations did not show any significant associations between the rate of filled pauses and standardized language scores. These results indicate that CHH produces *uh* more often than CTH, suggesting that they may have difficulty retrieving lexical–semantic items during ongoing speech. This information may be useful for interventionists who are collecting language samples during assessment.

## 1. Introduction

The primary aim of the current study is to investigate the use of filled pauses in child language samples, both as a function of hearing status (children with hearing loss and children with typical hearing) and as a function of language context (conversational language, expository discourse, and story retells). Filled pauses are a universal feature of language, similar to other disfluencies such as false starts, repetitions, and repairs, with *uh* and *um* being most common in the English language and *eh* being frequently used in other languages such as Swedish, Spanish, French, and Hebrew (Gorman et al., 2016; Irvine et al., 2016). Filled pauses can serve a number of functions such as expressing uncertainty and ambiguity (Brennan & Williams, 1995), planning utterances (Clark & Fox Tree, 2002), or word finding (Goodwin & Goodwin, 1986). Filled pauses can also serve as a linguistic cue to a communication partner; for example, speakers use these fillers to “hold the floor” in conversation (Maclay & Osgood, 1959) or indicate introduction of new information into a conversation (Arnold et al., 2003; Kidd et al., 2011) or a major syntactic boundary (Bailey & Ferreira, 2003; Martin & Strange, 1968; Swerts, 1998).

### 1.1. Discourse Functions of Filled Pauses Uh and Um

Recent literature has suggested that there are distinct functions for the use of *um* and *uh* during speech (Gorman et al., 2016; Irvine et al., 2016; McGregor & Hadden, 2020; Sun & Ding, 2025; Wehrle et al., 2024). The pattern of these findings indicates that the filled pause *uh* indicates minor delays during speech and serves the speaker’s needs by marking lexical and semantic retrieval. For example, *uh* occurs when a word that is being produced is low in frequency or contextual probability (Arnold et al., 2007). On the other hand, *um* appears to serve as a listener-oriented cue, marking that the speaker is holding the floor while they are gathering their thoughts, expressing uncertainty and ambiguity, or signifying another kind of pragmatic message they would like to convey to the listener. Researchers have posited that this listener-oriented function for *um* is supported by findings that *um* more often occurs at the start of a phrase, signals longer delays in speech and is more likely to be accompanied by a silent pause compared to *uh* (Clark & Fox Tree, 2002; Fox Tree, 2001; Sun & Ding, 2025).

Much of the prior work on the differing functions of *um* and *uh* as filled pauses has been conducted in populations with atypical language acquisition, particularly children with autism spectrum disorder (ASD) and developmental language disorders (DLD). Irvine et al. (2016) compared the use of filled pauses in spontaneous speech for three groups: children with ASD who had difficulties with pragmatic language, children with ASD who had “optimal outcomes” (i.e., had been previously diagnosed with ASD but demonstrated age-appropriate social and communication skills at the time of testing), and typically developing children. Participants ranged in age from 8 to 21 years. The language elicitation task required participants to describe six unrelated pictures, presented sequentially on a computer screen, while simultaneously tapping a key with their index finger as quickly as possible. The finger-tapping task served to increase the cognitive demand of the language elicitation task. In addition, each picture description was limited to 10 s to increase the task difficulty. Results showed no difference in *uh* rates across the three groups. In contrast, *um* rates were lower in the children with ASD who had pragmatic difficulty, while the ASD group with “optimal outcomes” produced *um* at a rate similar to that of the typically developing children. Children with greater autism severity showed a lower rate of *um*, but there was no correlation with executive functioning skills or structural language skills. These findings reinforce that *um* may be a pragmatic, listener-directed cue because children with pragmatic language deficits used *um* less frequently than children without pragmatic deficits. At the same time, the conclusions that can be drawn from this study are limited because the elicitation task involved a monolog rather than a dialog between two conversational partners. Thus, there is a gap in our knowledge regarding the use of filled pauses across different language contexts, particularly dialogic contexts.

Gorman et al. (2016) sought to determine whether filled pauses would distinguish children with ASD from children with DLD or typically developing children. In contrast to Irvine et al. (2016), who only used a picture-description task, Gorman and colleagues used multiple elicitation tasks, including a play-based language sample, picture description, telling a story from a book, and a conversational language sample. They also targeted a younger group of children, including 4- to 8-year-old children compared to the 8- to 21-year-olds in Irvine et al. Consistent with the results in Irvine et al. (2016), children in the ASD group used *um* significantly less often than typically developing children but did not differ from children with DLD. Typically developing children and children with DLD also did not differ in their use of *um*. There were no group differences between the three groups in terms of *uh* rate. The authors also reported a significant main effect of language elicitation task, but no interaction between group type and language task. The filled pause *uh* occurred the most often in conversational samples, followed by play-based activities, then picture description, and finally telling a story from a book. For *um*, conversations again had the highest rate of occurrence. The rate of *um* in the picture description and play-based sample occurred significantly less often than in conversation but were not significantly different from one another. Finally, telling a story from a book showed the lowest rate of *um* productions. The authors concluded that conversational interactions reduce one’s ability to plan and monitor ongoing speech, resulting in a higher cognitive load and higher use of filled pauses across all three groups in the study.

McGregor and Hadden (2020) used yet another type of language elicitation procedure, expository discourse, to replicate the findings of Irvine et al. (2016) and Gorman et al. (2016). Their sample included a group of 7- to 15-year-old with ASD and their age-matched typically developing peers. The expository discourse task required the participants to describe their favorite game or sport, including information on why it was their favorite game and a description of the rules of the game to a research assistant. There were no time limits or additional cognitive load in the expository discourse task. Similarly to the prior work, children with ASD used *um* less frequently than their typically developing peers but did not show a difference in *uh* rate.

Taken together, the results of these three studies suggest that filled pauses serve important discourse functions and may also help to distinguish children with atypical language acquisition from typically developing groups. To date, however, almost all of the research investigating the use of filled pauses in spontaneous speech has focused on children with ASD. There have been few to no studies examining other populations of children who may experience subtle difficulties with linguistic processing and planning or word retrieval. One such population, which is the focus of the current study, are children who are hard of hearing (CHH).

### 1.2. Children Who Are Hard of Hearing

Childhood hearing loss is a relatively common health condition, impacting approximately 11% of children and adolescents (Humes, 2024). Approximately 69% of the pediatric hearing loss population have hearing thresholds in the mild to severe range, also referred to as being “hard of hearing” (CDC, 2020). Historically, CHH demonstrated substantial delays in vocabulary, phonology, and grammar (Ching et al., 2013; Davis et al., 1986). Since the advent of early hearing detection and intervention and improvements in hearing technology, however, many school-age CHH perform within the average range on standardized language measures (Halliday et al., 2017; Tomblin et al., 2015a, 2015b, 2020a, 2020b). At the same time, there is also evidence to suggest that CHH who are scoring within the average range on norm-based assessments may continue to have difficulty with higher-level language tasks such as producing complex grammar (Nittrouer & Lowenstein, 2021; Vachio et al., 2023; Werfel & Douglas, 2017; Werfel et al., 2021). In addition, CHH may be slower to activate lexical and semantic representations in real time (Klein et al., 2023) or they may show deficits in lexical–semantic organization (Jerger et al., 2002, 2013; de Hoog et al., 2015). CHH also display slower growth in vocabulary depth (how much one knows about words), even when they are showing equivalent performance to hearing peers on measures of vocabulary breadth (how many words one knows) (Walker et al., 2019). Given that use of the filled pause *uh* has been linked to difficulties with lexical retrieval (Arnold et al., 2007), it is possible that CHH could also exhibit increased rates of *uh* compared to their hearing peers that are connected with their lexical–semantic difficulties. Cognitive processing is another area of weakness, particularly with phonological short-term memory, verbal working memory, and attention (Hansson et al., 2007; Heinrichs-Graham et al., 2022; McCreery & Walker, 2022; Stiles et al., 2012). Finally, there is some mixed evidence to suggest that CHH may have challenges with social-pragmatic aspects of language. For example, children who are deaf or hard of hearing have been shown to demonstrate inappropriate use of pragmatic functions (e.g., Most et al., 2010), but socio-pragmatic deficits also appear to be less significant or age-appropriate in children with mild-severe hearing loss than children with severe-profound hearing loss (Bongioletti et al., 2024; Tuohimaa et al., 2025). Given their documented deficits in higher-level lexical–semantic, and cognitive domains, it is plausible that some CHH will experience difficulty with planning and delivering speech in real time, and these difficulties may manifest in a higher rate of filled pauses compared to their typically hearing peers. To our knowledge, the hypothesis that CHH produces more filled pauses than their hearing peers has not been tested thus far.

### 1.3. The Current Study

To summarize, filled pauses are omnipresent in spoken interactions, serving an important role in the flow of conversations. The two most common types of filled pauses in English—*um* and *uh*—appear to have functionally distinct purposes during discourse, with *um* functioning as a listener-oriented conversational signal and *uh* functioning as a self-directed cue related to problems with finding words or formulating an utterance (Clark & Fox Tree, 2002). The current study seeks to test the hypothesis that CHH will produce different rates of fillers compared to their hearing peers. We propose that this differential rate is potentially the result of weaknesses in linguistic processing or lexical–semantic access for children with inconsistent auditory-linguistic access due to hearing loss.

Our research questions are as follows:

1. Do CHH and their peers with typical hearing differ in their use of *um* and *uh*?

Hypothesis: CHH will use *uh* more frequently than children with typical hearing (CTH), due to difficulty in lexical–semantic retrieval. We expect no significant differences in production of *um*.

2. Will children differ in the use of *um* and *uh* depending on discourse contexts?

Hypothesis: Children will use *uh* and *um* more often on tasks that require dialogic interactions (i.e., conversation > expository = fable retell).

3. Is variation in the use of filled pauses associated with language ability in CTH and CHH?

Hypothesis: CHH and CTH with weaker vocabulary/grammar skills will use filled pauses more frequently due to difficulties with lexical–semantic retrieval.

## 2. Materials and Methods

### 2.1. Participants

One hundred and one children participated in this study as part of a multicenter, longitudinal project focused on characterizing outcomes in children with hearing loss (Outcomes of Children with Hearing Loss Consortium [OCHLCON]), which took place between 2013 and 2018. Sixty-eight CHH (39 males) and 33 CTH (10 males) participated in the summer after completing fourth grade. Table 1 shows demographic characteristics of the participants. CHH were recruited via recommendations from service providers (e.g., audiologists, speech-language pathologists) or from Early Hearing Detection and Intervention registries. To be included in the OCHLCON study, CHH had to have a permanent, bilateral sensorineural, conductive, or mixed hearing loss, use spoken English as their primary communication mode, and have no additional significant cognitive or motor disabilities. The lack of additional disabilities was determined via parent report and service provider report. CTH also had to use spoken English as their primary communication mode and have no additional disabilities, as well as pass a 4-frequency hearing screening at 20 dB HL. Both CHH and CTH were assessed on nonverbal cognitive abilities as part of the overall test battery, which involved administration of the Wechsler Abbreviated Scale of Intelligence-Second Edition (WASI-II; Wechsler & Hsaio-Pin, 2011). All children were within at least 1.5 standard deviation of the norm-referenced mean on the WASI-II nonverbal subtests (i.e., Matrix Reasoning and Block Design).

CHH and CTH were matched on maternal education in years and chronological age in years (all *p*-values > 0.20; see Table 1). As seen in Table 1, while both the CHH and CTH are well-matched on maternal education level (which is a proxy for socioeconomic status), the mean maternal education level in both groups was around 16 years (i.e., at least a college degree). Sixty-two percent and 68% of the mothers of the CHH and CNH, respectively, had at least a four-year college degree. The demographic data indicate that both groups of research participants came from higher socioeconomic backgrounds than the average U.S. population, as the 2016 census (the midway point for when these data were collected) showed that 33.7% of women greater than 25 years had completed a four-year bachelor’s degree (Educational Attainment of the Population 18 Years and Over, by Age, Sex, Race and Hispanic Origin: 2016. US Census Bureau. Retrieved 29 July 2025). Seventy-nine percent (*n* = 54/68) of CHH had better-ear PTAs in the mild-moderate hearing loss range (less than 60 dB HL); the remaining 14 children had better-ear PTAs in the moderately severe to severe hearing loss range (60 to 80 dB HL). No children had a better-ear PTA in the profound hearing loss range (greater than 80 dB). Sixty-five percent (*n* = 44/68) had their hearing loss identified during the newborn hearing screening, while 35% were identified after they were newborns.

### 2.2. Procedures

Testing took place at either a university or clinical laboratory setting, a quiet location near the family (e.g., library), or in a research van that was designed to be a mobile testing unit.

#### 2.2.1. Audiologic Assessment and Hearing Aid Verification

For CHH, a pediatric audiologist obtained air-conduction and bone-conduction thresholds at 250, 500, 1000, 2000, 4000, 6000, and 8000 Hz. The four-frequency better-ear PTA at 500, 1000, 2000, and 4000 Hz was calculated for subsequent analyses. CTH completed a four-frequency hearing screening at 500, 1000, 2000, and 4000 Hz.

For CHH who wore HAs, the audiologist checked that the HAs were functioning appropriately based on manufacturers’ specifications (ANSI, 2003, S3.22). We also verified the audibility (i.e., amount of access to the long-term average speech spectrum) of the HAs by calculating the Speech Intelligibility Index (SII; ANSI, 1997, S3.5). The SII is a weighted numerical estimate of audibility across the speech frequency range. It is determined by measuring the sensation level of an average speech signal relative to an individual child’s hearing thresholds interpolated into one-third octave bands. Each octave band is weighted based on the contribution of said band to the average speech recognition score for adult listeners. The sensation level of each band is multiplied by each octave band’s importance weight. The final step in the SII calculation is that the weighted audibility of all bands is added together. This sum results in a value between 0 and 1, where 1 represents full audibility and 0 means no audibility. Using this approach, the audiologist performed probe microphone measures to quantify the real-ear-to-coupler difference (Bagatto et al., 2005, 2016) and then calculated aided SII based on user’s settings with a standard male speech signal (carrot passage; Cox & McDaniel, 1989) presented at average levels (60 or 65 dB SPL). From there, we determined the better-ear SII (BESII) for each participant with HAs.

#### 2.2.2. Standardized Language Assessments

Examiners administered two standardized language tests to assess vocabulary and grammar skills. The vocabulary measure consisted of the Woodcock Johnson Tests of Achievement-III (WJTA-III; Woodcock et al., 2007) Picture Vocabulary test. This measure assesses expressive vocabulary. Children were instructed to label pictured objects from a test booklet. Norm-referenced scores are reported as standard scores with a mean of 100 and a standard deviation of 15. The grammar measure consisted of the Clinical Evaluation of Language Fundamentals-4 (CELF-4; Semel et al., 2004) Formulated Sentences subtest. This measure assesses expressive grammar skills. The examiner presented a target word and a picture, and the children were instructed to produce a grammatically correct sentence that included the target word and described the picture. To keep language measures on the same scale, the CELF-4 Formulated Sentences scores were converted to standard scores with a mean of 100 and a standard deviation of 15.

#### 2.2.3. Language Sample

Participants engaged in three discourse activities: conversation, expository discourse, and fable retell. These three tasks were selected because each taps into different real-world communication contexts that children will encounter, both socially and academically. The first two activities—the conversational language sample and the expository discourse—followed protocols outlined in Nippold et al. (2005). Specifically, participants engaged in conversational discourse with an examiner who asked general questions about their family and media interests (e.g., describe your favorite book or movie). The conversational sample lasted approximately 5 to 8 min. The goal of the conversational sample was to engage the child in a dialogic interaction with the examiner. Second, participants engaged in an expository discourse task in which the child was asked to describe the rules of a game or sport. The examiner asked specific questions regarding what the child’s favorite game or sport was, why it was their favorite game or sport, and what strategies are required to win in that game or sport. The expository discourse requires higher syntactic complexity than conversational discourse because the speaker must communicate on topics with which the communication partner is (presumably) not familiar. At the same time, it is a monologic task that is relatively planned and structured and does not require conversational management skills such as turn-taking (Hadley, 1998). The third task involved a fable retell, following the protocol described in Nippold et al. (2017). Participants completed a fable retell in which the child was asked to retell a story, “The Fox and the Crow” in their own words after the examiner read it aloud to them once. The story retell task was presented with a single picture to elicit the fable. Similarly to expository discourse, the fable retell requires a high level of syntactic complexity in order to sound coherent, but has fewer pragmatic demands compared to dialogic, conversational interactions. As the fable task is a narrative retell, it also has higher memory demands compared to other types of language samples (Walker et al., 2023). The full protocol for the language sample is included in Appendix A.

Language samples were transcribed using Systematic Analysis of Language Transcripts (SALT) software (Heilmann & Miller, 2023). Two research assistants coded the language samples for filled pauses *um* and *uh*. Both research assistants were blind to the hearing status of the participants. Filled pauses were divided by total word count, resulting in *uh* and *um* ratio scores. Reliability was calculated for 10% of the samples. Intraclass correlation (ICC) analyses indicated excellent reliability: ICC (9, 10) r = 0.96 for *um*; ICC (9, 10) r = 0.99 for *uh*.

### 2.3. Statistical Analyses

Our first two research questions involved analyzing the effects of hearing status (hearing loss, typical hearing) and language discourse context (conversational, expository discourse, fable retell) on our two dependent variables: the ratios of *um* and *uh* usage in the language samples. We used a Generalized Estimating Equation (GEE) approach to address these research questions (Liang & Zeger, 1986) using SAS V 9.4 PROC GEE. This approach was selected over a repeated measures analysis of variance (ANOVA) or a linear mixed model approach because the outcome measures were highly right-skewed. A GEE approach is semi-parametric and therefore the full distribution is not specified for the analysis. We used an exchangeable working correlation structure to account for the correlation due to repeated measures and report results from the sandwich estimator of the standard error.

To address the third research question involving the associations between filled pauses and language abilities for CTH and CHH together, we used non-parametric Spearman’s tests. The independent variables were standard scores on the WJTA Picture Vocabulary test and CELF-4 Formulated Sentences. Again, our rationale behind this non-parametric approach was to account for the high level of skewness in the outcome data.

## 3. Results

### 3.1. Standardized Language Assessments

As shown in Table 1, CHH had significantly lower vocabulary scores on the Woodcock Johnson Tests of Achievement-III (WJTA) Picture Vocabulary Test (*p* = 0.04, Cohen’s d = 0.45) and expressive syntax scores on the CELF-4 Formulated Sentences subtest (*p* = 0.04; Cohen’s d = 0.42) compared to the CTH, who were matched on age and maternal education level. It should also be noted that the mean standard scores for both language assessments were within the average range for the CHH, and slightly above average for the CTH. As noted in the methods section, however, both groups of children had mean maternal education levels that were higher than the average U.S. population. Thus, the control group of CTH is a more accurate comparison for determining language delays than the normative sample of the standardized tests, which are not matched on socio-economic background with the CHH in the current study.

### 3.2. Effects of Hearing Status and Language Discourse Context on Filled Pauses

Table 2 displays the medians, means, and standard deviations for the *um* and *uh* ratios across all language discourse contexts as a function of hearing status. In the GEE models, we did not find a significant interaction between hearing status and language discourse context for *um* or *uh*. Therefore, we removed the interaction term from the analyses and examined the main effects for the fixed factors.

Our first research question asked whether CHH would differ in their use of *uh* or *um* compared to CTH. We hypothesized that CHH would use *uh* more frequently than CTH, but we would not see significant differences in *um*. This hypothesis was confirmed: in terms of hearing status, CHH and CTH differed significantly in their use of *uh*, z = −2.43, *p* = 0.015 with CHH producing *uh*-filled pauses at a significantly higher ratio across the language sample contexts than their peers with typical levels of hearing. CHH and CTH did not differ in their use of *um*, z = 0.80, *p* = 0.4265.

Our second research question asked whether children would differ in their use of filled pauses as a function of language discourse context. We hypothesized that children would use filled pauses more often on tasks that required dialogic interactions (i.e., conversations > expository discourse = fable retell). Our hypothesis was confirmed for *um*-filled pauses but not *uh*-filled pauses. First, we found a main effect of context for *um* and *uh* (X2[2] =16.81, p = 0.0002 and X2[2] = 6.14, p = 0.0465, respectively). Follow-up pairwise comparison between contexts using a Tukey–Kramer adjustment indicated that *um* was produced more often in conversational samples compared to expository discourse (*p* = 0.0070) and fable retell (*p* = 0.0010), but there was no significant difference between expository and fable retell (*p* = 0.2018). *Uh* was produced relatively more often in conversational samples compared to expository discourse (*p* = 0.0367) but there was no significant difference between conversational samples and fable retell (*p* = 0.4851) or expository discourse and fable retell (*p* = 0.8837).

**Figure 1:**
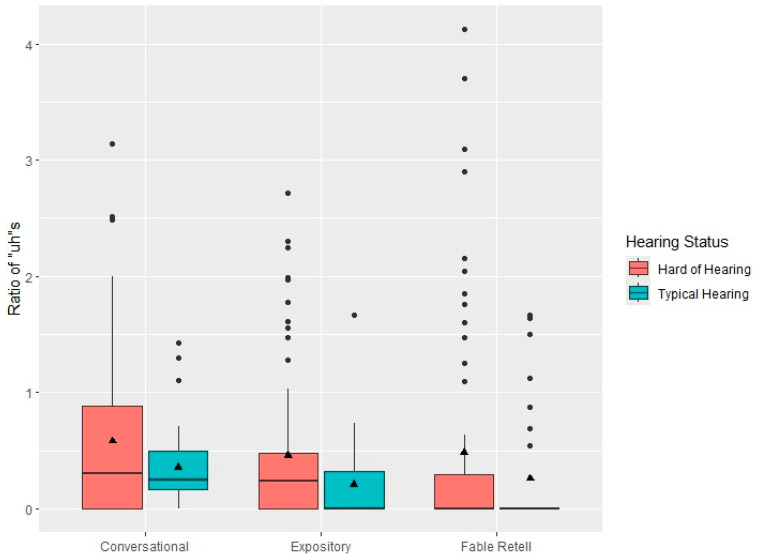
Boxplots of conversational samples (**left**), expository discourse (**middle**), and fable retell (**right**) filled pause *uh* rates as a function of hearing status. The central lines represent the median values, the filled triangles within the boxplots represent the mean values, and the box limits are the 25th and 75th percentiles. The filled circles outside the box plots represent outliers.

**Figure 2:**
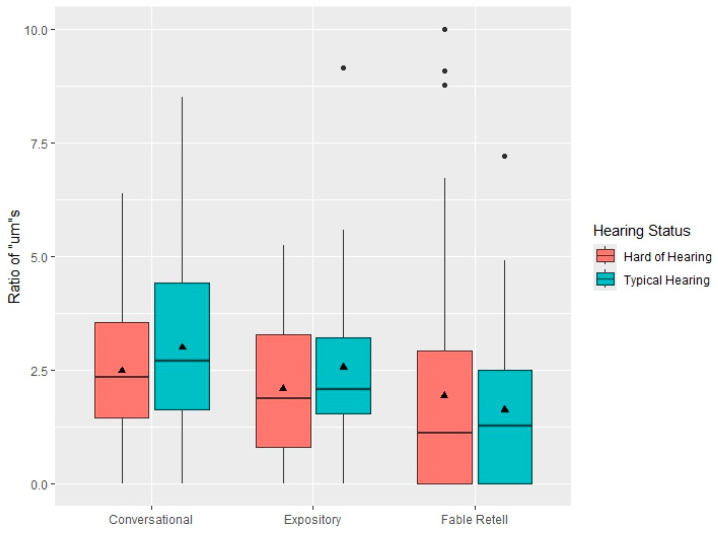
Boxplots of conversational samples (**left**), expository discourse (**middle**), and fable retell (**right**) filled pause *um* rates as a function of hearing status. The central lines represent the median values, the filled triangles within the boxplots represent the mean values, and the box limits are the 25th and 75th percentiles. The filled circles outside the box plots represent outliers.

Figure 1 displays box plots for the *uh* ratios in the three language contexts separated by hearing status. As seen in Table 1 and Figure 1, the means and medians for *uh* ratios in the CHH are higher than the CTH, across all language discourse contexts. Across both CHH and CTH, the mean and median uh ratios are higher in the conversational samples than the expository discourse. Figure 2 displays box plots for *um* ratios, which illustrates the higher mean and median ratios in the conversational language samples relative to the expository discourse or fable retells for both the CHH and CTH. Although the box plots appear to show a trend in which the mean and median *um* ratios are higher for the CTH than the CHH in the conversational and expository discourse contexts, the statistical analysis did not indicate a significant difference between groups.

### 3.3. Associations Between Filled Pauses and Language Abilities

Our third research question asked whether variation in filled pauses is associated with language abilities. We had hypothesized that children with weaker vocabulary/grammar skills would produce filled pauses at a higher rate than children with stronger vocabulary/grammar skills. This hypothesis was not confirmed: we found no significant associations between *um* or *uh* ratios in the three language sample contexts and expressive vocabulary scores on the WJ-III Picture Vocabulary test or expressive grammar on the CELF-4 Formulated Sentences (all *p*-values > 0.05).

## 4. Discussion

The primary objective of the current study was to compare the use of filled pauses in CHH and CTH across different language contexts. We hypothesized that CHH would use *uh* more often than CTH due to their linguistic and cognitive processing limitations in complex linguistic tasks. We did not expect to see differences between groups in terms of *um*-filled pauses given a lack of strong evidence that CHH have a primary deficit in the social-pragmatic domain. We further hypothesized that we would see differences in the rate of filled pauses for both *uh* and *um* based on the language elicitation procedure, with filled pauses occurring more often in dialogic conversational tasks compared to expository discourse or story retells. Finally, we hypothesized that the rate of filled pauses would be associated with individual differences in language abilities, in which children who had weaker vocabulary or morphosyntactic skills would use filled pauses more frequently than children with stronger vocabulary or morphosyntactic skills.

Overall, our results were consistent with two of our three hypotheses. CHH used *uh* significantly more often compared to CTH; we speculate that they may have had difficulty with lexical–semantic retrieval, resulting in more self-directed cues during spontaneous speech. There were no significant differences in use of *um* between groups, which could indicate appropriate use of listener-directed cues for CHH. As predicted, we also saw an effect of discourse type, with both *uh* and *um* being used more often in dialogic, conversational interactions than expository discourse, which are more monologic tasks with less cognitive load than conversations. Inconsistent with our third hypothesis, we did not find an association with the rate of filled pauses and language skills.

### 4.1. Effect of Hearing Status on the Rate of Filled Pauses

There is a fairly robust literature on the use of filled pauses in children with atypical language acquisition, but almost all of those prior studies have focused on children with primary deficits in social communication, specifically children with ASD (Gorman et al., 2016; Irvine et al., 2016; MacFarlane et al., 2017; McGregor & Hadden, 2020; Parish-Morris et al., 2017). To the best of our knowledge, no studies have examined the use of filled pauses in children with hearing loss, making the results of the current study a novel contribution to the literature on the use of fillers in populations with atypical language acquisition.

In previous research, a consistent finding across is that children with ASD tended to use *um* less frequently than children with typical language acquisition (or children with developmental language delays), and there were no group differences in the rate of *uh*. The filler *um* is thought to be a specific social-communication signal, serving a pragmatic role in conversations (e.g., as a cue for politeness and attention to the conversational partner, or an indication of the length and informativeness of future speech). Children with ASD tend to have difficulty taking a conversational partner’s perspective into account (Paul et al., 2009). Thus, they may produce *um* at a lower rate than their peers without ASD because they are less likely to use a listener-centered linguistic cue in ongoing spontaneous speech.

Children who are deaf (i.e., profound hearing loss) have been shown to have some weaknesses in pragmatic skills (for a review, see Paul et al., 2020) but there is less research on pragmatic conversational skills in CHH. The research that is available suggests that children with better unaided hearing tend to have stronger pragmatic skills than children with more severe-profound levels of hearing loss (Tuohimaa et al., 2025). The current findings would suggest that CHH produce *um* as a listener-oriented cue at the same rate as their hearing peers. This might indicate that pragmatic skills, and the ability to take a conversational partner’s needs into account, is a relative strength for CHH. Unfortunately, a limitation of the current study is that we did not conduct any formal assessments of pragmatic language skills, such as the Pragmatics Checklist (Goberis et al., 2012) or the Pragmatic Protocol (Prutting & Kittchner, 1987). Further research is needed to characterize pragmatic language use in children who have hearing loss in the mild to severe range. In addition, we hesitate to make any strong inferences about a lack of a difference based on hearing status, given that we are underpowered to draw any firm conclusions that the two groups are equivalent in their use of *um* in spontaneous speech.

In contrast to the findings on *um* productions, we did see a significant difference in the rate of *uh* productions as a function of hearing status. The children with hearing loss used the *uh*-filled pause at a significantly higher rate than their hearing peers, who were matched on both age and socioeconomic status to the CHH. In typical language acquisition, the *uh* variant frequently occurs in the middle of utterances rather than the beginning and is usually a signal for a short delay in ongoing speech (Fox Tree, 2001; Swerts, 1998). It is often used during repairs or self-corrections (Brennan & Schober, 2001). Prior studies have shown that CHH who have language delays have challenges with word retrieval and lexical–semantic organization (de Hoog et al., 2015; Esbensen & Thomsen, 2021; Marshall et al., 2018; Rush et al., 2023), as well as cognitive processing difficulties related to attention and verbal short-term and working memory (Hansson et al., 2007; McCreery & Walker, 2022). Therefore, we suggest that the higher rate of *uh* productions among the CHH in the current study could reflect their difficulties in retrieving lexical–semantic items from long-term memory, particularly when the participants encountered the real-time demands of discourse. At the same time, we acknowledge that this suggestion is highly speculative, and additional research is needed to replicate and substantiate the current findings.

### 4.2. Effect of Language Context on the Rate of Filled Pauses

Language sampling is an important and ecologically valid approach for capturing a child’s language abilities (Werfel & Douglas, 2017). There are a number of ways to elicit spontaneous language and different discourse types can have varying effects on language sampling outcomes (Hadley, 1998; Spencer et al., 2023). The current study utilized a language sampling protocol with three different forms of discourse: conversational sampling, expository discourse, and a story retell (Nippold, 2013). Previous studies have used a single type of discourse to evaluate the rate of filled pauses; for example, Lake et al. (2011) collected conversational samples from adults with and without ASD, while Irvine et al. (2016) elicited speech in a picture description task and McGregor and Hadden (2020) used the same expository discourse task as the current study (i.e., favorite game or sport) in children. Gorman et al. (2016) conducted one of the few studies to compare the use of fillers across multiple discourse contexts. They used tasks from the Autism Diagnostic Observation Schedule (ADOS) to elicit language during conversation, play-based activities, picture description, and telling a story from a book. Our results replicated those of Gorman et al., in that we found that discourse context influenced filled pause rates, with *um* ratios being higher for conversational samples relative to the other two types of discourse. *Uh* ratios were also significantly higher in the conversational samples compared to the expository discourse, although there were no differences in the rates of *uh* between the conversational samples and fable retells. Conversational interactions, particularly with unfamiliar listeners, require higher planning demands than monologic expository discourse or story retells, and increased planning demands result in elevated disfluencies (Bortfeld et al., 2001). Based on the current findings and previous studies, we see a robust effect of discourse context on filled pause rates. Researchers and clinicians who are interested in examining filled pauses should be cognizant of the linguistic and cognitive demands of the elicitation procedure they are using, particularly when exploring group differences in typical and atypical language learners (McGregor & Hadden, 2020).

### 4.3. Individual Differences in Filled Pauses

Contrary to our hypothesis, we did not find that language ability was correlated with filled pause rates. This lack of an effect on individual differences in filled pause rates may have been due to the norm-referenced measures that were used in the analysis, in that end-point expressive language measures may have not sufficiently tapped into the real-time linguistic processing deficits that CHH demonstrated. Irvine et al. (2016) also examined possible underlying mechanisms for fillers and did not find an association between executive functions or general language abilities on *um* rates, although they did find that ASD severity was negatively associated with filler production. Future studies should more thoroughly investigate the underlying factors driving variation in filled pauses, to better understand the mechanisms that are associated with disfluent speech.

### 4.4. Limitations

One important limitation of this study is that all participants used spoken English as their primary communication mode. While fillers appear to be nearly universal across languages and dialects, they can take different forms in languages other than English, particularly in terms of the vowel quality. Furthermore, languages other than West Germanic languages (e.g., tonal languages) may use different types of disfluencies more frequently than filled pauses (Wehrle et al., 2024). Thus, the results of the current study may not generalize to other languages. An interesting future direction of this work would be to examine the use of filled pauses in languages other than English.

A second limitation of the current work is that all of the participants with hearing loss had bilateral hearing levels in the mild to severe range, and no participants had a profound hearing loss in both ears. We also did not have any participants with cochlear implants in the study. Given the limited knowledge surrounding verbal disfluencies and hesitations in children with any degree of hearing loss, it would be beneficial to replicate this study with a group of children who have greater degrees of hearing loss.

## 5. Conclusions

Taken together, the major findings of this study indicate that CHH use significantly more *uh* fillers in discourse activities compared to CTH and the rate of filled pauses varies based on discourse context. Although the CHH in this study performed within the average range on standardized language assessments, they scored significantly lower on those measures and showed a difference in their use of the filled pause *uh* relative to hearing peers who were matched on socio-economic status. These results highlight how norm-referenced assessments may be insufficient as primary sources of evidence for language delays in CHH and it is important to utilize both local norms and dynamic assessments when assessing children with potential language learning difficulties (Werfel & Douglas, 2017). In summary, the current results fill a gap in our knowledge of the effects of congenital or early-onset hearing loss and discourse context on filled pauses in spontaneous speech. This study also highlights the importance of using different types of language samples as an assessment tool when evaluating school-age children.

## Figures and Tables

**Table 1:** Demographic and audiological characteristics and test scores for children who are hard of hearing (CHH) and children with typical hearing (CTH).

Test Variable	CHH Mean (SD)	CTH Mean (SD)	Between Group *p*-Values
Age (years)	10.38 (0.34)	10.33 (0.40)	*p* = 0.49
Maternal education level (years)	15.79 (2.65)	16.58 (3.27)	*p* = 0.21
WJTA-III Picture Vocabulary SS	99.48 (16.42)	114.80 (13.55)	*p* = 0.04 *
CELF-4 Formulated Sentences SS	104.12 (13.66)	110.30 (15.51)	*p* = 0.04 *
Better-ear PTA (dB HL)	45.36 (15.26)	<20	NA
Better-Ear Aided SII	78.72 (0.13)	NA	NA
Age at HL confirmation (months)	20.09 (21.56)	NA	NA
Age at HA fit (months)	23.55 (22.36)	NA	NA

Note. WJTA-III SS = Woodcock Johnson Tests of Achievement-III standard score; CELF-4 = Clinical Evaluation of Language Fundamentals-4 standard score; PTA = pure-tone average; SII = Speech Intelligibility Index; HL = hearing loss; HA = hearing aid, NA = not applicable. * indicates significance with alpha level = 0.05.

**Table 2:** Median, mean, and standard deviation for ratios of uh and um for conversational samples, expository discourse, and fable retell in children who are deaf or hard of hearing (CDHH) and children with typical hearing (CTH).

	CDHH			CTH		
Test Variable	Median	Mean	SD	Median	Mean	SD
Conversational *uh* ratio	0.31	0.58	0.73	0.24	0.36	0.36
Conversational *um* ratio	2.35	2.49	1.54	2.70	3.00	1.88
Expository *uh* ratio	0.23	0.46	0.67	0.00	0.21	0.34
Expository *um* ratio	1.87	2.09	1.51	2.08	2.55	1.75
Fable retell *uh* ratio	0.00	0.49	0.99	0.00	0.26	0.53
Fable retell *um* ratio	1.13	1.94	2.51	1.28	1.62	1.83

## Data Availability

The data sets generated and/or analyzed during this study are available from the corresponding author upon request.

## Abbreviations

The following abbreviations are used in this manuscript:

CHH	Children who are hard of hearing
CTH	Children with typical hearing

## Appendix A

EXPOSITORY DISCOURSE GENERATION

**4th GRADE PROTOCOL**

Follow the protocol below carefully, making sure that you perform **all** activities and ask **all** questions, even if (in passing) the child already answered a question.

NOTE: Scripts are in bold italics

1. General Conversation

***First of all, I’d like to learn something about you. I’m going to ask you a few general questions. Then you can ask me some questions, too. OK?***

A. ***Tell me about your family. Do you have any brothers or sisters? (if yes) What are their names? How old are they? What else can you tell me about them?***
B. ***Do you have any pets at home? (if yes) Tell me about your pets.***
C. ***Do you have a favorite TV show or movie? Tell me about it.***
D. ***Do you like to read books or magazines? (if yes) Which ones?***
E. ***Now do you want to ask me anything? (allow 1–2 quick questions)***
2. **Favorite Game or Sport Task**

***Let’s shift topics. I am hoping to learn what people know about certain games and sports. So now I’d like to ask you some different questions.***

If the child is reluctant to talk, offer encouraging comments; re-present the questions as necessary. If the child answers in one or two word responses, repeat what the child says, show interest and say, “That’s interesting, can you tell me anything else?” or “Can you tell me more about it, something about it, etc.?” You can prompt as often as needed. If it is clear the child cannot say more, move on. There is no set amount of talking time that we are aiming for.

**What is your favorite game or sport? (pause…)**

**Why is (checkers/baseball) your favorite game/sport? (pause…)**

***I’m not too familiar with the game/sport of (checkers/baseball), so I would like you to tell me all about it. For example, tell me what the goals are, and how many people may play a game/match.***

***Now please tell me about the rules the players need to follow. Tell me everything you can think of about the game/sport of (checkers/baseball) so that someone who has never played it before will know how to play.***

Now I would like you to tell me what a player should do in order to win the game/sport of (checkers/baseball). In other words, what are some key strategies that every good player should know?

3. **Story Retelling Task**

***This is the final activity for this task.***

***This is a story telling activity that involves a fable. Fables are imaginary stories about animals that act like people. I am going to read you a fable. Please listen carefully and be ready to tell it back to me, in your own words. Try to remember as much as you can so that you can tell the whole story back to me. After you finish, I will ask you some questions about the story. Are you ready?***

- SHOW FABLE PICTURE TO CHILD

***The title of this fable is, “The Fox and the Crow”…***

***A crow flew to the top of a tree to enjoy a piece of cheese she had stolen. A wily fox spied the cheese in the crow’s beak, and his mouth watered for it, as he spoke these words:***

***“O beautiful crow, charming crow, delightful crow, how wonderful to see you in all your glory!”***

***The crow smiled, holding the cheese firmly in her beak.***

***“You are the most exquisite of birds—and though I have not heard your voice, I am quite sure it must surpass that of any other bird.”***

***The vain crow was pleased by all this flattery, especially the part about her voice, for she had sometimes been told that her caw was unpleasant. She opened her beak to sing for the fox, and out fell the cheese into the fox’s watering jaws.***

***Moral: “Beware of flattery”***

***Now I’d like you to tell the story back to me, in your own words. Try to tell me everything you can remember about the story…. (pause)***

## References

[B1-behavsci-15-01053] ANSI (1997). American National Standards methods for the calculation of the Articulation Index *(S3.5-1997 R-2007)*.

[B2-behavsci-15-01053] ANSI (2003). Specifications of hearing aid characteristics *(S3.22–2003)*.

[B3-behavsci-15-01053] Arnold J. E., Fagnano M., Tanenhaus M. K. (2003). Disfluencies signal theee, um, new information. Journal of Psycholinguistic Research.

[B4-behavsci-15-01053] Arnold J. E., Kam C. L. H., Tanenhaus M. K. (2007). If you say thee uh you are describing something hard: The on-line attribution of disfluency during reference comprehension. Journal of Experimental Psychology: Learning, Memory, and Cognition.

[B5-behavsci-15-01053] Bagatto M., Moodie S., Brown C., Malandrino A., Richert F., Clench D., Scollie S. (2016). Prescribing and verifying hearing aids applying the American academy of audiology pediatric amplification guideline: Protocols and outcomes from the ontario infant hearing program. Journal of the American Academy of Audiology.

[B6-behavsci-15-01053] Bagatto M., Moodie S., Scollie S., Seewald R., Moodie S., Pumford J., Liu K. R. (2005). Clinical protocols for hearing instrument fitting in the desired sensation level method. Trends in Amplification.

[B7-behavsci-15-01053] Bailey K. G., Ferreira F. (2003). Disfluencies affect the parsing of garden-path sentences. Journal of Memory and Language.

[B8-behavsci-15-01053] Bongioletti J., Doble M., Purcell A. (2024). Conversation and pragmatics in children who are hard-of-hearing: A scoping review. Journal of Deaf Studies and Deaf Education.

[B9-behavsci-15-01053] Bortfeld H., Leon S. D., Bloom J. E., Schober M. F., Brennan S. E. (2001). Disfluency rates in conversation: Effects of age, relationship, topic, role, and gender. Language and Speech.

[B10-behavsci-15-01053] Brennan S. E., Schober M. F. (2001). How listeners compensate for disfluencies in spontaneous speech. Journal of Memory and Language.

[B11-behavsci-15-01053] Brennan S. E., Williams M. (1995). The feeling of another′s knowing: Prosody and filled pauses as cues to listeners about the metacognitive states of speakers. Journal of Memory and Language.

[B12-behavsci-15-01053] Centers for Disease Control and Prevention (CDC) (2020). Data and statistics about hearing loss in children.

[B13-behavsci-15-01053] Ching T. Y., Dillon H., Marnane V., Hou S., Day J., Seeto M., Crowe K., Street L., Thomson J., Van Buynder P., Zhang V. (2013). Outcomes of early-and late-identified children at 3 years of age: Findings from a prospective population-based study. Ear and Hearing.

[B14-behavsci-15-01053] Clark H. H., Fox Tree (2002). Using uh and um in spontaneous speaking. Cognition.

[B15-behavsci-15-01053] Cox R. M., McDaniel D. M. (1989). Development of the speech intelligibility rating (SIR) test for hearing aid comparisons. Journal of Speech, Language, and Hearing Research.

[B16-behavsci-15-01053] Davis J. M., Elfenbein J., Schum R., Bentler R. A. (1986). Effects of mild and moderate hearing impairments on language, educational, and psychosocial behavior of children. Journal of Speech and Hearing Disorders.

[B17-behavsci-15-01053] de Hoog B. E., Langereis M. C., van Weerdenburg M., Knoors H., Verhoeven L. (2015). Lexical access in children with hearing loss or specific language impairment, using the cross-modal picture–word interference paradigm. Research in Developmental Disabilities.

[B18-behavsci-15-01053] Esbensen A., Thomsen P. (2021). Word retrieval and lexical organization in children with hearing loss and developmental language disorder. Communication Disorders Quarterly.

[B19-behavsci-15-01053] Fox Tree J. E. (2001). Listeners’ uses of um and uh in speech comprehension. Memory & Cognition.

[B20-behavsci-15-01053] Goberis D., Beams D., Dalpes M., Abrisch A., Baca R., Yoshinaga-Itano C. (2012). The missing link in language development of deaf and hard of hearing children: Pragmatic language development. Seminars in Speech and Language.

[B21-behavsci-15-01053] Goodwin M. H., Goodwin C. (1986). Gesture and coparticipation in the activity of searching for a word. Semiotica.

[B22-behavsci-15-01053] Gorman K., Olson L., Hill A. P., Lunsford R., Heeman P. A., van Santen J. P. (2016). Uh and um in children with autism spectrum disorders or language impairment. Autism Research.

[B23-behavsci-15-01053] Hadley P. A. (1998). Language sampling protocols for eliciting text-level discourse. Language, Speech, and Hearing Services in Schools.

[B24-behavsci-15-01053] Halliday L. F., Tuomainen O., Rosen S. (2017). Language development and impairment in children with mild to moderate sensorineural hearing loss. Journal of Speech, Language, and Hearing Research.

[B25-behavsci-15-01053] Hansson K., Sahlén B., Mäki-Torkko E. (2007). Can a ‘single hit’cause limitations in language development? A comparative study of Swedish children with hearing impairment and children with specific language impairment. International Journal of Language & Communication Disorders.

[B26-behavsci-15-01053] Heilmann J., Miller J. F. (2023). Systematic analysis of language transcripts solutions: A tutorial. Perspectives of the ASHA Special Interest Groups.

[B27-behavsci-15-01053] Heinrichs-Graham E., Walker E. A., Eastman J. A., Frenzel M. R., McCreery R. W. (2022). Amount of hearing aid use impacts neural oscillatory dynamics underlying verbal working memory processing for children with hearing loss. Ear and Hearing.

[B28-behavsci-15-01053] Humes L. (2024). Audiograms and prevalence of hearing loss in US children and adolescents 6–19 years of age. Journal of Speech, Language, and Hearing Research.

[B29-behavsci-15-01053] Irvine C. A., Eigsti I. M., Fein D. A. (2016). Uh, um, and autism: Filler disfluencies as pragmatic markers in adolescents with optimal outcomes from autism spectrum disorder. Journal of Autism and Developmental Disorders.

[B30-behavsci-15-01053] Jerger S., Martin R. C., Damian M. F. (2002). Semantic and phonological influences on picture naming by children and teenagers. Journal of Memory and Language.

[B31-behavsci-15-01053] Jerger S., Tye-Murray N., Damian M. F., Abdi H. (2013). Effect of hearing loss on semantic access by auditory and audiovisual speech in children. Ear and Hearing.

[B32-behavsci-15-01053] Kidd C., White K. S., Aslin R. N. (2011). Toddlers use speech disfluencies to predict speakers’ referential intentions. Developmental Science.

[B33-behavsci-15-01053] Klein K. E., Walker E. A., McMurray B. (2023). Delayed lexical access and cascading effects on spreading semantic activation during spoken word recognition in children with hearing aids and cochlear implants: Evidence from eye-tracking. Ear and Hearing.

[B34-behavsci-15-01053] Lake J. K., Humphreys K. R., Cardy S. (2011). Listener vs. speaker-oriented aspects of speech: Studying the disfluencies of individuals with autism spectrum disorders. Psychonomic Bulletin & Review.

[B35-behavsci-15-01053] Liang K.-Y., Zeger S. L. (1986). Longitudinal data analysis using generalized linear models. Biometrika.

[B36-behavsci-15-01053] MacFarlane H., Gorman K., Ingham R., Presmanes Hill A., Papadakis K., Kiss G., Van Santen J. (2017). Quantitative analysis of disfluency in children with autism spectrum disorder or language impairment. PLoS ONE.

[B37-behavsci-15-01053] Maclay H., Osgood C. E. (1959). Hesitation phenomena in spontaneous english speech. Word.

[B38-behavsci-15-01053] Marshall C. R., Jones A., Fastelli A., Atkinson J., Botting N., Morgan G. (2018). Semantic fluency in deaf children who use spoken and signed language in comparison with hearing peers. International Journal of Language & Communication Disorders.

[B39-behavsci-15-01053] Martin J. G., Strange W. (1968). The perception of hesitation in spontaneous speech. Perception & Psychophysics.

[B40-behavsci-15-01053] McCreery R. W., Walker E. A. (2022). Variation in auditory experience affects language and executive function skills in children who are hard of hearing. Ear and Hearing.

[B41-behavsci-15-01053] McGregor K. K., Hadden R. R. (2020). Brief report: “Um” fillers distinguish children with and without ASD. Journal of Autism and Developmental Disorders.

[B42-behavsci-15-01053] Most T., Shina-August E., Meilijson S. (2010). Pragmatic abilities of children with hearing loss using cochlear implants or hearing aids compared to hearing children. The Journal of Deaf Studies and Deaf Education.

[B43-behavsci-15-01053] Nippold M. A. (2013). Explaining complex matters: How knowledge of a domain drives language. Expository discourse in children, adolescents, and adults.

[B44-behavsci-15-01053] Nippold M. A., Hesketh L. J., Duthie J. K., Mansfield T. C. (2005). Conversational versus expository discourse. Journal of Speech, Language, and Hearing Research.

[B45-behavsci-15-01053] Nippold M. A., Vigeland L. M., Frantz-Kaspar M. W., Ward-Lonergan J. M. (2017). Language sampling with adolescents: Building a normative database with fables. American Journal of Speech-Language Pathology.

[B46-behavsci-15-01053] Nittrouer S., Lowenstein J. H. (2021). When language outgrows them: Comprehension of ambiguous sentences in children with normal hearing and children with hearing loss. International Journal of Pediatric Otorhinolaryngology.

[B47-behavsci-15-01053] Parish-Morris J., Liberman M. Y., Cieri C., Herrington J. D., Yerys B. E., Bateman L., Donaher J., Ferguson E., Pandey J., Schultz R. T. (2017). Linguistic camouflage in girls with autism spectrum disorder. Molecular Autism.

[B48-behavsci-15-01053] Paul R., Orlovski S. M., Marcinko H. C., Volkmar F. (2009). Conversational behaviors in youth with high-functioning ASD and Asperger syndrome. Journal of Autism and Developmental Disorders.

[B49-behavsci-15-01053] Paul R., Paatsch L., Caselli N., Garberoglio C. L., Goldin-Meadow S., Lederberg A. (2020). Current research in pragmatic language use among deaf and hard of hearing children. Pediatrics.

[B50-behavsci-15-01053] Prutting C. A., Kittchner D. M. (1987). A clinical appraisal of the pragmatic aspects of language. Journal of Speech and Hearing Disorders.

[B51-behavsci-15-01053] Rush O., Werfel K. L., Lund E. (2023). Lexical–semantic organization as measured by repeated word association in children who are deaf and hard of hearing who use spoken language. Journal of Speech, Language, and Hearing Research.

[B52-behavsci-15-01053] Semel E., Wiig E., Secord W. (2004). Clinical evaluations of language fundamentals-4.

[B53-behavsci-15-01053] Spencer T. D., Tolentino T. J., Foster M. E. (2023). Impact of discourse type and elicitation task on language sampling outcomes. American Journal of Speech-Language Pathology.

[B54-behavsci-15-01053] Stiles D. J., Bentler R. A., McGregor K. K. (2012). The speech intelligibility index and the pure-tone average as predictors of lexical ability in children fit with hearing aids. Journal of Speech, Language, and Hearing Research.

[B55-behavsci-15-01053] Sun Y., Ding H. (2025). Pausing patterns in English school-age children with a history of late talking: Frequent pauses and prolonged response delays. Journal of Communication Disorders.

[B56-behavsci-15-01053] Swerts M. (1998). Filled pauses as markers of discourse structure. Journal of Pragmatics.

[B57-behavsci-15-01053] Tomblin J. B., Harrison M., Ambrose S. E., Walker E. A., Oleson J. J., Moeller M. P. (2015a). Language outcomes in young children with mild to severe hearing loss. Ear and Hearing.

[B58-behavsci-15-01053] Tomblin J. B., Oleson J., Ambrose S. E., Walker E. A., McCreery R. W., Moeller M. P. (2020a). Aided hearing moderates the academic outcomes of children with mild to severe hearing loss. Ear and Hearing.

[B59-behavsci-15-01053] Tomblin J. B., Oleson J., Ambrose S. E., Walker E. A., Moeller M. P. (2020b). Early literacy predictors and second-grade outcomes in children who are hard of hearing. Child Development.

[B60-behavsci-15-01053] Tomblin J. B., Walker E. A., McCreery R. W., Arenas R. M., Harrison M., Moeller M. P. (2015b). Outcomes of children with hearing loss: Data collection and methods. Ear and Hearing.

[B61-behavsci-15-01053] Tuohimaa K., Loukusa S., Löppönen H., Aarnisalo A. A., Dietz A., Hyvärinen A., Laitakari J., Rimmanen S., Salonen J., Sivonen V., Tennilä T. (2025). Factors associated with social-pragmatic understanding in deaf and hard of hearing and typically hearing 6-year-old children. Journal of Speech, Language, and Hearing Research.

[B62-behavsci-15-01053] Vachio M., Lund E., L. Werfel K. (2023). An analysis of mental state verb and complex syntax use in children who are deaf and hard of hearing. Language, Speech, and Hearing Services in Schools.

[B63-behavsci-15-01053] Walker E. A., Harrison M., Baumann R., Moeller M. P., Sorensen E., Oleson J. J., McCreery R. W. (2023). Story generation and narrative retells in children who are hard of hearing and hearing children. Journal of Speech, Language, and Hearing Research.

[B64-behavsci-15-01053] Walker E. A., Redfern A., Oleson J. J. (2019). Linear mixed-model analysis to examine longitudinal trajectories in vocabulary depth and breadth in children who are hard of hearing. Journal of Speech, Language, and Hearing Research.

[B65-behavsci-15-01053] Wechsler D., Hsaio-Pin C. (2011). Wechsler abbreviated scale of intelligence-second edition (WASI-II).

[B66-behavsci-15-01053] Wehrle S., Grice M., Vogeley K. (2024). Filled pauses produced by autistic adults differ in prosodic realisation, but not rate or lexical type. Journal of Autism and Developmental Disorders.

[B67-behavsci-15-01053] Werfel K. L., Douglas M. (2017). Are we slipping them through the cracks? the insufficiency of norm-referenced assessments for identifying language weaknesses in children with hearing loss. Perspectives of the ASHA Special Interest Groups.

[B68-behavsci-15-01053] Werfel K. L., Reynolds G., Hudgins S., Castaldo M., Lund E. A. (2021). The production of complex syntax in spontaneous language by 4-year-old children with hearing loss. American Journal of Speech-Language Pathology.

[B69-behavsci-15-01053] Woodcock R. W., McGrew K. S., Mather N. (2007). Woodcock Johnson III tests of achievement-normative update.

